# Editorial: Zoonotic Parasitic Diseases in a Changing World

**DOI:** 10.3389/fvets.2021.715112

**Published:** 2021-07-08

**Authors:** Serena Cavallero, Simona Gabrielli, Alessia Libera Gazzonis, Marco Pombi, Viliam Šnábel

**Affiliations:** ^1^Department of Public Health and Infectious Diseases, Sapienza University of Rome, Rome, Italy; ^2^Department of Veterinary Medicine, Università degli Studi di Milano, Milan, Italy; ^3^Institute of Parasitology, Slovak Academy of Science, Bratislava, Slovakia

**Keywords:** zoonoses, parasites, neglected diseases, global change, NTDs, neglected tropical diseases

Parasites are diverse and challenging group of eukaryotes, including zoonotic pathogens naturally occurring in the environment, already significantly changed by globalization and anthropogenic impact. Climate changes can further modify fundamental features and transmission dynamics of zoonosis (e.g., parasites' host preference, infectivity, areal). The proximity of humans and animals in several settings, such as in the rural landscapes, as well as in fragmented sylvatic habitats, in the natural environment close to urban areas and for the companion relationship between human and animals, may represent additional risk factors.

The Research Topic aims to gather the most updated studies on zoonotic neglected and foodborne parasites, taking into account two pivotal aspects: (i) challenging scenarios represented by climate change and anthropogenic impact and (ii) the “One-Health” concept.

The Research Topic collected 11 contributions including 3 reviews, 1 minireview, 1 brief research report and 6 original research articles, ranging from protozoans (*Toxoplasma, Leishmania, Cryptosporidium*) to metazoans (*Echinococcus, Taenia, Dirofilaria, Toxocara, Trichuris*).

## Evidences From Protozoans

Vector-borne diseases are particularly interesting in the global change scenario, given the susceptibility of vectors to climatic variations and their large plasticity and adaptive features to several ecological contexts. In the review of Springer et al., the authors called for filling the gap in knowledge to determine if and how global changes impact, in terms of climate, land use, agricultural practices and human behavior, the frequency of zoonotic tick-borne pathogens in domestic animals. Similarly, the presence of *Leishmania infantum* was investigated in a poorly studied host, i.e., cats, in the Mediterranean basin, a key area for leishmaniosis particularly for the growing tendency of tourists to travel with pets (Morelli et al.). Apart from the Mediterranean basin, the East African region remains one of the most globally impacted for leishmaniosis: Jones and Welburn underlined the necessity to implement surveillance and disease management measures, especially in low- and middle-income countries, expected to be most impacted by climate change (3).

Another protozoan disease affecting both humans and livestock, and having a huge impact in low-income countries, is cryptosporidiosis. Utaaker et al. highlighted the need to investigate epidemiological scenarios of cryptosporidiosis in goats, one of the most frequently bred species, especially in low-income countries. Despite the resulting negligible concern for zoonotic transmission in these areas, scant information is available for developing countries so far.

Finally, the role of two thioredoxin proteins from *Toxoplasma gondii* was studied in host-parasite interplay by the means of CRISPR-Cas9 (Zhang et al.). The study excluded their involvement as virulence factors *in vitro* and *in vivo* experiments. This study evidenced the application of an innovative approach such as the CRISPR-Cas9 system to investigate biological functions of parasitic proteins and host-parasite interactions.

## Evidences From Metazoans

Several metazoans are included in the Neglected Tropical Diseases (NTDs) list and the World Health Organization (WHO) has recently launched its road map “Ending the neglect to attain the Sustainable Development Goals: a road map for neglected tropical diseases 2021–2030,” as highlighted in the review about neurocisticercosis, a disease that represents a significant health burden to affected communities (Butala et al.).

Cystic and alveolar echinococcosis are two of the most important zoonotic parasites with public health and economic relevance, ranked in highest positions among the food-borne zoonoses according to the international food safety agencies. Their presence in wild and domesticated animals still pose a challenge for the disease control. In the study of Dehghani et al., the genetic diversity of systematically relevant genomic region of *E. granulosus* was evaluated in samples from camels, which play a significant role in the parasite transmission cycle, especially in the Middle East and North Africa (7). By comparing the haplotypes obtained in the sample with homologous sequences from all over the world, the researchers observed a moderate genetic variation occurring in the sample, showing *E. intermedius* (G6) as the most commonly represented genotype in camels, followed by *E. granulosus* sensu stricto (G1). In addition, the Middle East isolates were more variable than the North/sub-Saharan isolates, suggesting an ancestrality for *E. granulosus* originating from the former region. The study offers a global outlook of the importance of camels in the molecular epidemiology of cystic echinococcosis in the main camel breeding areas of the world. Genetic diversity of the related species *E. multilocularis*, in which four distinct clades corresponding approximately to their continents of origin are described, was recently investigated (Umhang et al.). With molecular epidemiology, it is possible also to trace various records of genetic variants in atypical regions, which may be the result of introduction or natural migration of host animals. In this scenario, red foxes from Eastern Europe (Poland) harbor haplotypes of the typical European *E. multilocularis*, but also of the Asian cluster. The study presented the first large-scale investigation showing a zone of apparent overlap between European and Asian variants of *E. multilocularis*. Because the resolution from microsatellite EmsB analyzes and mitochondrial (mt) sequencing did not completely agree on allocation to Asian and European clusters, the authors suggested that cross-fertilization between worms of Asian origin and worms from the European Polish population might explain these conflicting results.

Regarding the phylum Nematoda, the role of microRNAs (miRNAs) was investigated in the infection progression caused by *Toxocara canis*, a neglected zoonotic parasite, which threatens the health of dogs and humans worldwide (Zou et al.). Considering the understudied and fascinating role of miRNas in host-parasite interplay, particularly in chronic infections induced by nematodes, this study is of great relevance for a better understanding of the adaptive and innate immune responses mechanisms associated with dog infections by *T. canis*.

Riveiro et al. examined the current knowledge of *Trichuris trichiura* and its relationship to whipworm of other primates. This study contributes to the debate on the systematics of *T. trichiura*, in updating data supporting the existence of several taxonomic entities with different degree of host affiliation circulating in primates.

The epidemiological status of the cardiopulmonary dirofilariosis caused by *Dirofilaria immitis* in dogs and humans is updated for the Colombian region (Esteban-Mendoza et al.) reporting a considerable seropositivity in both dogs (around 11%) and humans (around 7%), indicating comparable transmission rates also in atypical hosts as humans.

Thanks to a broader and more comprehensive view of interacting factors characterizing a zoonotic disease, the scientific community could better understand major elements that contribute to the fundamental aspects of parasite transmission and how they are closely linked to the distribution and persistence of these diseases. This article collection emphasize how interdisciplinary collaboration is becoming an essential feature of epidemiological surveys, being of paramount importance in developing effective strategies for the control of zoonotic diseases.

## Author Contributions

This editorial was authored by SC, with review and input by SG, VS, AG, and MP. All authors contributed to the article and approved the submitted version.

## Conflict of Interest

The authors declare that the research was conducted in the absence of any commercial or financial relationships that could be construed as a potential conflict of interest.

